# Short Term Usage of Omega-3 Polyunsaturated Fatty Acids Ameliorate Lipopolysaccharide-Induced Inflammatory Response and Oxidative Stress in the Neonatal Rat Hippocampal Tissue

**DOI:** 10.3389/fnut.2020.572363

**Published:** 2020-11-17

**Authors:** Jipeng Shi, Weiwei wang, Guimei Sang, Huifang Xi, Yazhou Sun, Chaosheng Lu, Hezhen Ye, Limi Huang

**Affiliations:** ^1^Henan Key Laboratory of Neurorestoratology, Department of Neonatology, The First Affiliated Hospital of Xinxiang Medical University, Weihui, China; ^2^The First Affiliated Hospital of Wenzhou Medical University, Zhejiang, Wenzhou, China; ^3^The Second Affiliated Hospital of Wenzhou Medical University, Zhejiang, Wenzhou, China

**Keywords:** omega-3 polyunsaturated fatty acids, lipopolysaccharide, proinflammatory cytokines, oxidative stress, neonatal rat brain

## Abstract

**Objective:** To investigate the effect of omega-3 polyunsaturated fatty acids (ω-3 PUFAs) on lipopolysaccharide (LPS)-induced inflammatory response and oxidative stress in neonatal rat brain.

**Methods:** Ninety-six 3-day-old Sprague Dawley rats were divided into four groups: control (saline/saline), LPS/ω-3, LPS/ω-6, and LPS/saline (*n* = 24/group). All rats, except those in the control group, were intraperitoneally challenged once with LPS (0.6 mg/kg) and were treated with ω-3 PUFAs, ω-6 PUFAs, or saline at 15 mL/kg for 1 or 5 consecutive days beginning on the day of LPS-challenge. Rats in the control group underwent the same procedures and received saline (vehicle). After 1 or 5 days of treatment, 12 rats from each group were sacrificed and their hippocampuses were collected. The expression of inflammation-related genes as well as the levels of oxidative stress markers in hippocampal tissues were determined.

**Results:** After 1 or 5 days of treatment, the expression of toll-like receptor 4 and multiple proinflammatory cytokines were significantly decreased in the LPS/ω-3 group compared with those in the LPS/saline group. The activities of superoxide dismutase and glutathione (GSH) were significantly elevated, whereas amounts of malondialdehyde and oxidized glutathione (GSSG) and the ratio of GSSG/GSH were remarkably lowered in the LPS/ω-3 group compared with those in the LPS/saline group after 1 day of treatment. Opposite effects were observed in the LPS/ω-6 group.

**Conclusion:** ω-3 PUFAs may protect rat brain tissue against LPS-induced inflammatory response and oxidative stress.

## Introduction

Preterm birth, a leading cause of neonatal mortality, remains an important public health challenge worldwide. Approximately 15 million infants are born preterm each year, accounting for 11% of all pregnancies (1, 2). It is estimated that one-third of preterm birth survivors suffer from long-term neurological disabilities (3), such as palsy, mental retardation, epilepsy, which are majorly attributed to neonatal infection-induced cerebral injury (4). The most common form of brain injury in premature birth survivors is white matter injury (WMI), which is defined by degeneration of preoligodendrocytes (5). It is well-established that preoligodendrocytes are particularly vulnerable to oxidative stress and inflammation, which are the two major mechanisms underlying the injury and death of preoligodendrocytes (3, 6). Thus, targeting oxidative stress and inflammation, in conjunction with antimicrobial agents, is an important adjuvant strategy that could be employed to prevent or ameliorate WMI in neonatal infections (7–9).

Lipopolysaccharide (LPS), a major cell wall component of Gram-negative bacteria, has been widely used to model various brain diseases in rodents (10–13). As an inflammation inducer (14), LPS initiates inflammation through binding to toll-like receptor 4 (TLR4), which induces translocation of nuclear factor kappa B (NF-κB) into nuclei, leading to the release of various proinflammatory cytokines, including tumor necrosis factor-alpha (TNF-α), interleukin-1β (IL-1β), and IL-6 (15–17). In addition, LPS has been shown to induce oxidative damage in various diseases (18, 19). In causing brain disorders, LPS can stimulate reactive oxygen species (ROS) formation, resulting in significant alteration in the levels of superoxide dismutase (SOD), malondialdehyde (MDA), glutathione (GSH), and oxidized glutathione (GSSG) in the brain (13, 19). Previous studies have indicated that a single dose of systemic LPS is adequate to induce the production of inflammatory cytokines and alter the levels of oxidative stress markers in the prefrontal cortex and hippocampus of murine models (20–22).

Unlike saturated and monounsaturated fatty acids that can be synthesized in the liver, omega-3 (ω-3) and omega-6 (ω-6) polyunsaturated fatty acids (PUFAs) are considered essential FAs in the human diet derived, respectively, from alpha-linolenic acid (ALA, 18:3 ω-3) and linoleic acid (LA, 18:2 ω-6) (23). Both of them are routinely used as a part of intravenous nutrition in preterm infants to meet their nutritional needs (24). ω-3 PUFAs consisting of eicosapentaenoic acid (EPA) and docosahexaenoic acid (DHA) are primarily derived from fish oil. Dietary supplementation of ω-3 PUFAs has been found to exert anti-inflammatory effects against a number of inflammatory diseases, such as osteoarthritis and inflammatory bowel disease (25, 26). A previous study showed that ω-3 PUFAs can reduce the secretion of pulmonary inflammatory factors (TNF-α, IL-1β, and IL-6) in rats with acute lung injury through suppressing the TLR4/NF-κB signaling pathway. By contrast, ω-6 PUFAs, richer in the Western diet, comprise (conjugated) LA, gamma-linolenic acid, and arachidonic acid (ARA) (23). ARA is a precursor to a number of potent pro-inflammatory mediators including well described prostaglandins and leukotrienes (23). It is generally believed that ω-3 PUFAs lead to the production of anti-inflammatory eicosanoids, decosanoid neuroprotectins, and resolvins while the longer ω-6 fatty acid ARA tends to the generation of proinflammatory eicosanoids (leukotriene, prostaglandin and thromboxane) (27).

However, little is known about the effect of ω-3 PUFAs in infection-induced inflammation and oxidative stress in neonatal brain (28–30). In the present study, we examined and compared the effect of ω-3 PUFAs on LPS-induced inflammation and oxidative stress in the hippocampal tissue of 3-day-old neonatal rats (31). The expression of the TLR4/NF-κB signaling and downstream proinflammatory cytokines as well as the amounts of oxidative stress markers were measured in the hippocampal tissue. Our findings provided new insights into the potential benefit of ω-3 PUFAs in preventing and alleviating infection-triggered brain injury in preterm infants.

## Materials and Methods

### Animals

A total of 96 neonatal (3-day-old) Sprague Dawley (SD) rats were provided by and reared in the Center of Laboratory Animal Science at Xinxiang Medical university (Xinxiang, Henan, China). Rats maintained at 25°C under a 12-h light/12-h dark cycle were randomly divided into four groups: control (saline/saline), LPS/saline, LPS/ω-3, and LPS/ω-6 (*n* = 24/group). All rats, except those in the control group, were intraperitoneally (i.p.) injected with 0.6 mg/kg LPS (*Escherichia coli* serotype O111:B4, Sigma-Aldrich, USA) to induce inflammation and oxidative stress as previously described (17). The rats were either treated with sterile saline, 10% ω-3 PUFAs (100 mL contains: highly refined fish oil 10.0 g, EPA 1.25–2.82 g, DHA 1.44–3.09 g, myristic acid 0.1–0.6 g, palmitic acid 0.25–1.0 g, palmitoleic acid 0.3–0.9 g, stearic acid 0.05–0.2 g, oleic acid 0.6–1.3 g, linoleic acid 0.1–0.7 g, linolenic acid ≤ 0.2 g, octadecatetraenoic acid 0.05–0.4 g, eicosaenoic acid 0.05–0.3 g, ARA 0.1–0.4 g, docosaenoic acid ≤ 0.15 g, docosapentaenoic acid 0.15–0.45 g, dl-α-Tocopherol 0.015–0.0296 g), or 20% ω-6 PUFAs [Sigma-Aldrich, USA, 100 mL contains: soy bean oil 10 g (content of essential fatty acids: Linoleic acid 4.38–5.86 g; α-Linolenic acid 0.45–1.1 g); Glycerol 2.5 g; Phospholipids from egg 0.6 g] *via* i.p. injection at 15 mL/kg/day for 1 or 5 consecutive days, beginning on the day of LPS-challenge. Rats in the control group underwent the same procedure and received the same volume of saline as vehicle (29). Twelve rats in each group were chosen randomly and sacrificed at 1 or 5 days after treatment, respectively. The hippocampal tissues were immediately collected, weighed, and stored at −80°C until use. This study was approved by and all animal procedures were conducted in accordance with the Animal Care and Use Committee of Health Guide for the Care and Use of Laboratory Animals.

### Polymerase Chain Reaction (RT-PCR)

Total RNA was extracted from the hippocampal tissue using TRIzol reagent (Invitrogen, Carlsbad, CA, USA) according to the manufacturer's instructions. One μg of total RNA was converted to cDNA using a reverse transcription kit (Promega, Madison, WI, USA, No. A3500) following the manufacturer's instructions. PCR amplification was carried out using Taq DNA polymerase (Takara, Tokyo, Japan) in a thermal cycler (580BR, Bio-Rad, Hercules, CA, USA). The primers (Table 1) were designed and synthesized by Sangon Biological Engineering Technology & Services Co., Ltd. (Shanghai, China) based on sequences from GenBank. Each reaction started at 95°C for 5 min, amplified with 35 cycles of 30 s at 94°C, 30 s at the annealing temperature, and 60 s at 72°C, and ended with 10 min of extension at 72°C. The annealing temperatures for TLR4, NF-κB, TNF-α, IL-1β, IL-6, and GAPDH (β-actin) were 55, 61, 60, 65.5, 60, and 51°C, respectively. Then, 7.5 μL of each PCR product was subject to electrophoresis on a 1% agarose gel, and the density of each band was in a double-blinded manner analyzed on a gel image analysis system. The mRNA level of TLR4, NF-κB, TNF-α, IL-1β, or IL-6 was respectively normalized and determined based on the following density relative to the β-actin mRNA.

**Table 1:** PCR primers used in the study.

**Gene**	**Primer sequences**
TLR4	F:5′-ACAGGGCACAAGGAAGTAGC-3′
	R:5'-GTTCTCACTGGGCCTTAGCC-3′
NF-κB	F: '-CATACGCTGACCCTAGCCTG-3'
	R:5′-TTTCTTCAATCCGGTGGCGA-3′
TNF-α	F:5′-CCAACAAGGAGGAGAAGT-3′
	R:5′-GTATGAAGTGGCAAATCG-3′
IL-1β	F: 5′-GCAACTGTCCCTGAACTCAACT-3′
	R:5′-TTGTCGAGATGCTGCTGTGA-3′
IL-6	F: 5′- AACGATGATGCACTTGCAGA-3′
	R:5′-GGAAATTGGGGTAGGAAGGA-3′
GAPDH	F:5′-GGCACAGTCAAGGCTGAGAATG-3′
	R:5′-ATGGTGGTGAAGACGCCAGTA-3′

### Western Blot Analysis

Protein lysates were obtained from hippocampal tissue using a protein extract kit (Active Motif, Tokyo, Japan) according to the manufacturer's protocols. Thirty micrograms proteins were separated on 7.5% SDS-PAGE gel, and then transferred onto 0.45 μm PVDF membranes (Bio-Rad). The membranes were blocked with 5% nonfat milk in Tris-buffered saline containing 0.1% Tween 20 (TBST) for 2 h and then incubated overnight at 4°C with rabbit polyclonal antibody against mouse TLR4, NF-κB, TNF-α, IL-1β, or IL-6 (Cell Signaling Technology, USA). After washing with TBST 3 times at room temperature for 10 min each, the membranes were incubated with a horseradish peroxidase (HRP)-conjugated secondary antibody (1:2,000, Cell Signaling Technology) for 1.5 h. After 3 washes with TBST, immunoreactive bands were visualized with an electrochemiluminescence reagent (AmerControl, Uppsala, Sweden). GAPDH (Cell Signaling Technology, USA) were used as protein controls to normalize protein expression levels. Densitometric quantification of the protein bands was performed using Image Lab software (Bio-Rad).

### Biochemical Measurements

The amounts/enzymatic activity of SOD, MDA, GSSG, and GSH were determined using commercial kits (Jiancheng Bioengineering Institute, Nanjing, China), respectively; GSH and GSSG levels were also measured using additional kits from Cayman Chemical, USA.

### Statistical Analysis

Data were expressed as the mean ± standard deviation and analyzed using SPSS 20.0 for Windows (IBM, Armonk, NY, USA). Statistical analysis was carried out using one-way analysis of variance for each time point. Comparisons among groups were conducted using Tukey-Kramer, and verified by using Bonferroni *post-hoc* test with *P-*values expressed in the following results. A value of *P* **<** 0.05 was considered significant.

## Results

### ω-3 PUFAs Downregulate the Expression of Inflammation-Related Genes and Proteins in Neonatal Rat Hippocampal Tissues

**Figure 1:**
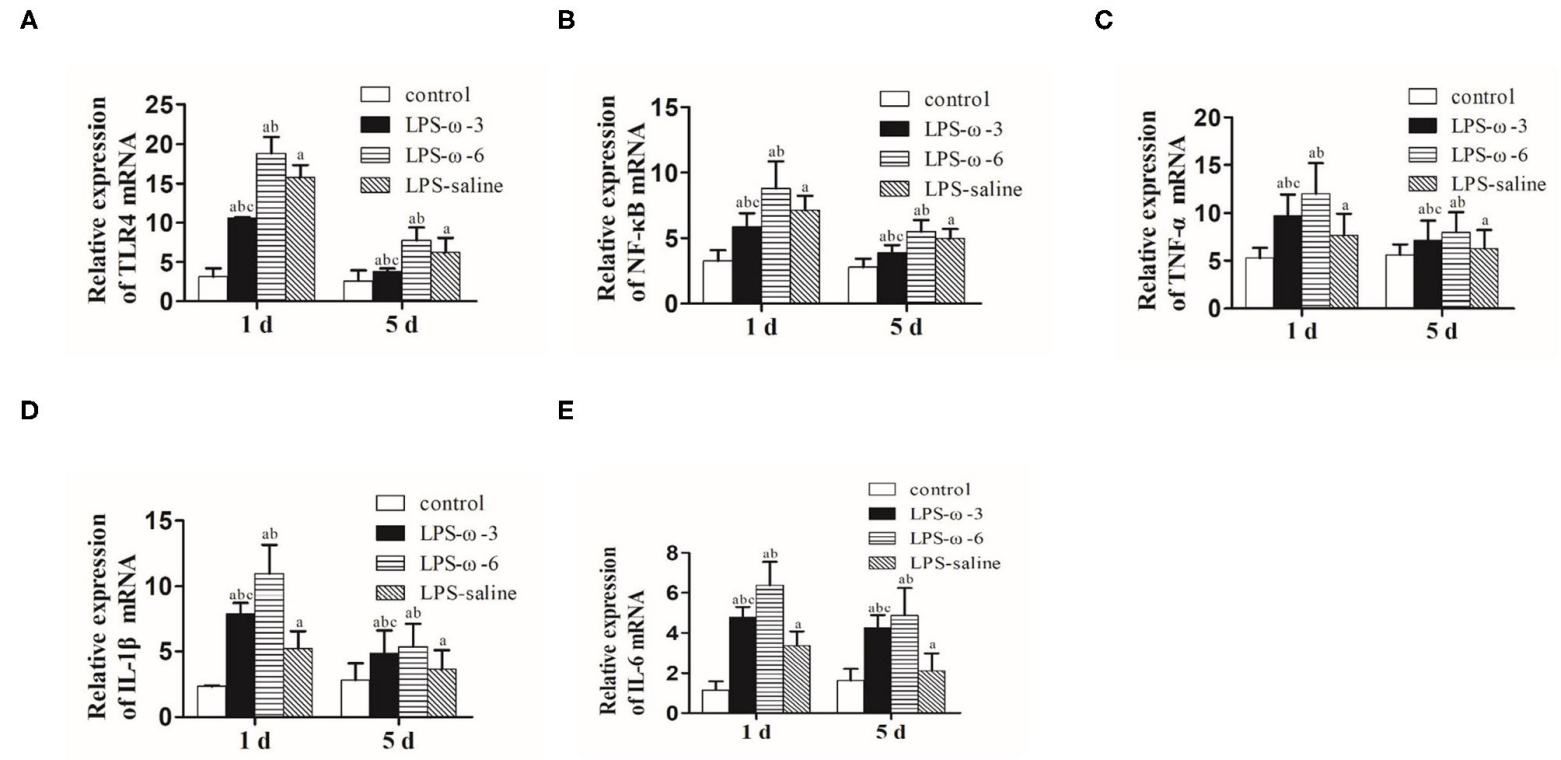
mRNA expression of inflammation-related genes. PCR was performed to determine the mRNA expression of TLR4 **(A)**, NF-κB **(B)**, TNF-α **(C)**, IL-1β **(D)**, and IL-6 **(E)** in the hippocampus of neonatal rats. Data are expressed as mean ± standard deviation (SD). ^a^*P* < 0.05 vs. the control group, ^b^*P* < 0.05 vs. the LPS group, ^c^*P* < 0.05 vs. the ω-6 group; *n* = 12. LPS, lipopolysaccharide; NF-κB, nuclear factor kappa B; TNF-α, tumor necrosis factor-α; IL-1β, interleukin-1β; IL-6, interleukin-6.

**Figure 2:**
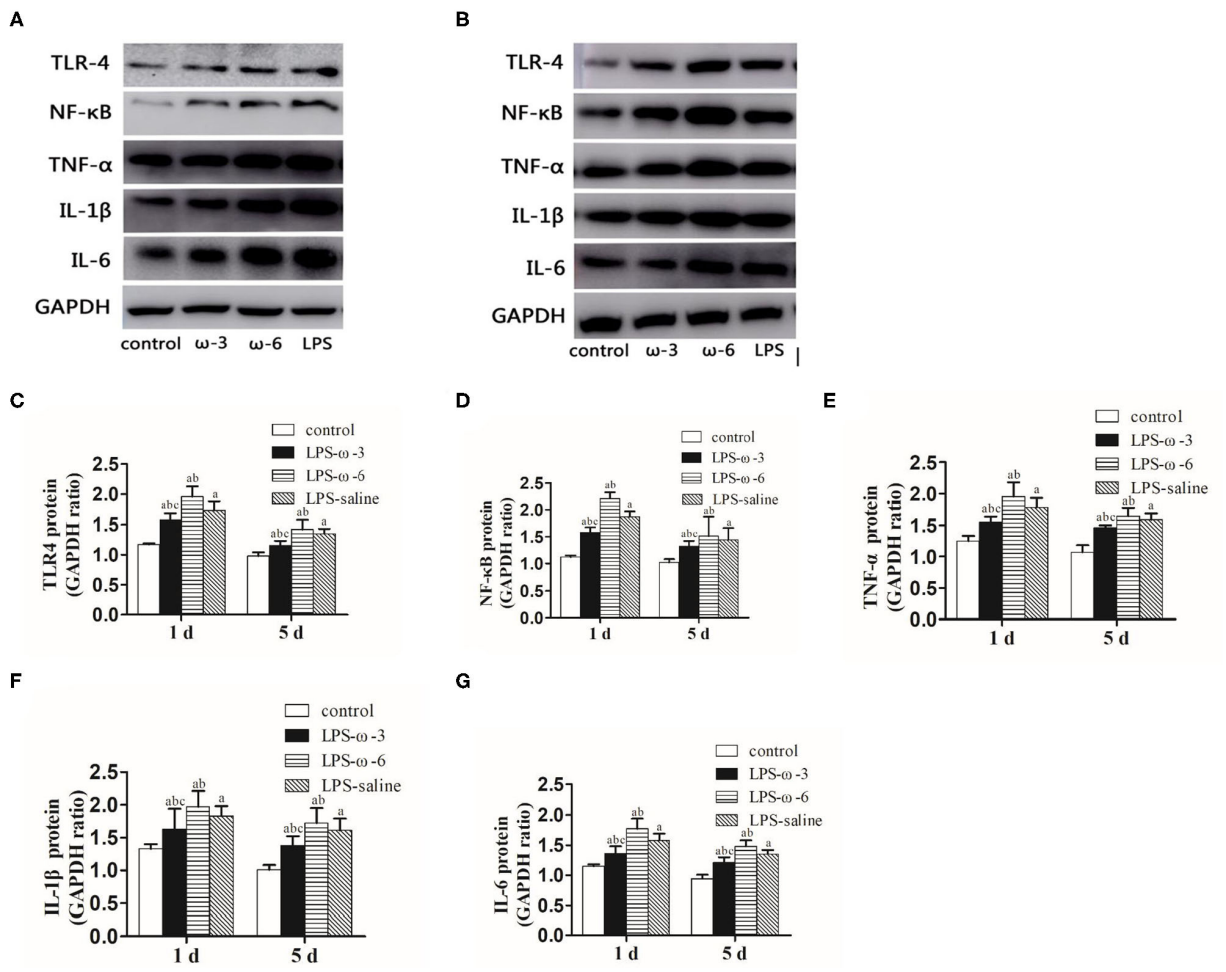
Protein expression of inflammation-related genes. **(A)** Western blot results showing the TLR4, NF-κB, TNF-α, IL-1β, and IL-6 proteins in the hippocampus in neonatal rats from the four groups 1 day after the intraperitoneal injection of drugs. **(B)** Western blot results showing the TLR4, NF-κB, TNF-α, IL-1β, and IL-6 proteins in the hippocampus in neonatal rats from the four groups 5 days after the intraperitoneal injection of drugs. Western blot analysis was performed to determine the protein expression of TLR4 **(C)**, NF-κB **(D)**, TNF-α **(E)**, IL-1β **(F)**, and IL-6 **(G)** protein expression in the hippocampus in neonatal rats from the different groups. The data points represent the mean ± SD; *n* = 12 (^a^*P* < 0.05 compared with the control group; ^b^*P* < 0.05 compared with the LPS group; ^c^*P* < 0.05 compared with the ω-6 group).

To determine whether ω-3 plays a role in the modulation of inflammation induced by LPS, we determined the expression of the TLR4/NF-κB signaling and downstream proinflammatory cytokines in hippocampal tissues of rats. As shown in Figure 1, LPS challenge unanimously and significantly increased the mRNA levels of TLR4 (A), NF-κB (B), TNF-α (C), IL-1β (D), and IL-6 (E) compared with the control group at both timepoints (day 1 or day 5 after treatment). Importantly, the mRNA levels of these inflammation-related genes were markedly decreased in the LPS/ω-3 group when compared with the LPS/saline group. By contrast, opposite effects were observed in the LPS/ω-6 group (Figures 1A–E). Similar trends were found in the protein levels of these genes (Figures 2A–G).

### ω-3 PUFAs Reduce the Alterations of Oxidative Stress Markers in Neonatal Rat Hippocampal Tissues

**Figure 3:**
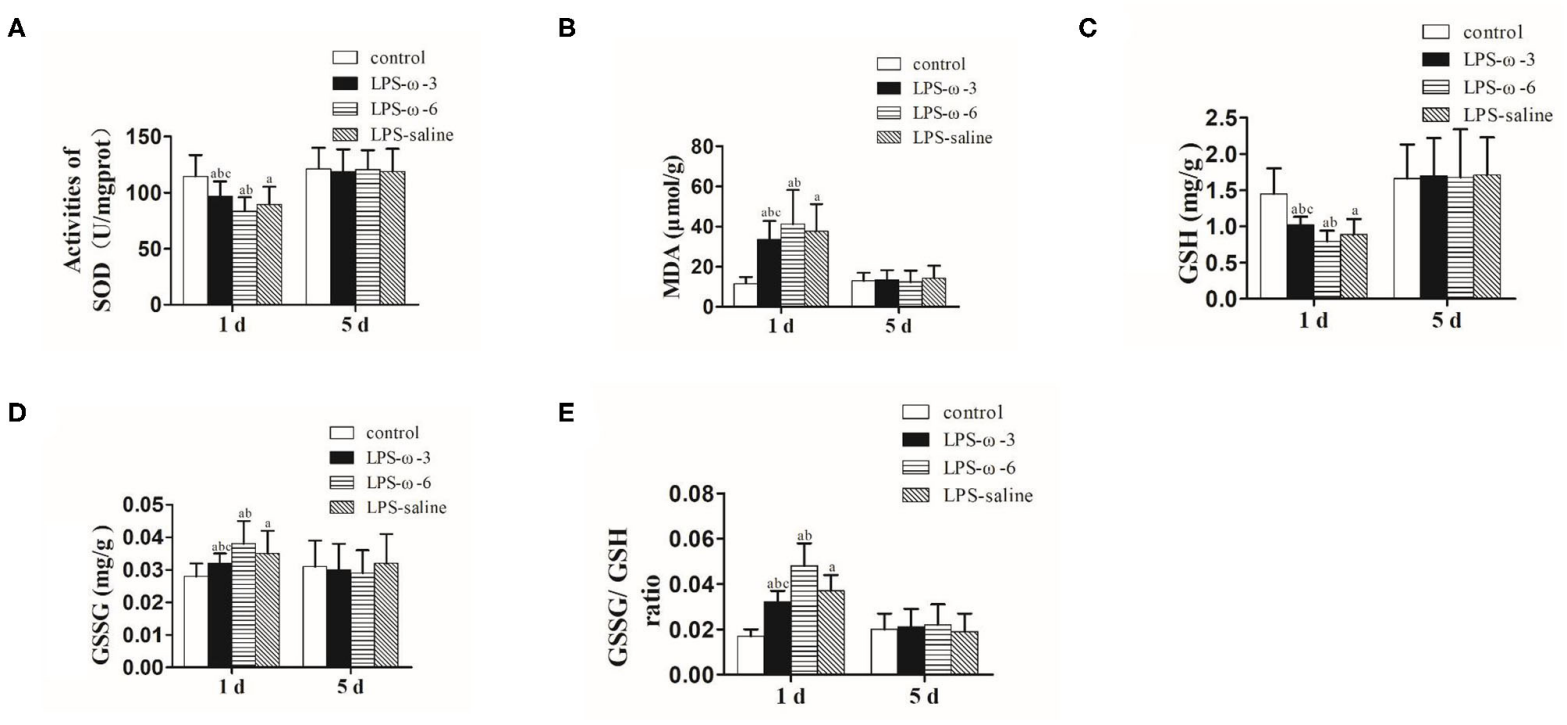
The amounts of SOD **(A)**, MDA **(B)**, GSH **(C)**, GSSG **(D)**, and GSSG/GSH **(E)** in the hippocampal tissues from the different groups. The data points represent the mean ± SD; *n* = 12 (^a^*P* < 0.05 compared with the control group; ^b^*P* < 0.05 compared with the LPS group; ^c^*P* < 0.05 compared with the ω-6 group). SOD, superoxide dismutase; MDA, malondialdehyde; GSH, glutathione; GSSG, oxidized glutathione.

To explore the effect of ω-3 PUFAs on LPS-induced oxidative stress, we measured the levels of oxidative stress markers in neonatal rat hippocampal tissues. As shown in Figure 3, ω-3 treatment significantly elevated the enzymatic activities of SOD (A) and GSH (C) but remarkably reduced the amounts of MDA (B) and GSSG (D) as well as the ratio of GSSG/GSH (E) compared to the LPS/saline group after 1 day of treatment. Opposite effects were observed in the LPS-ω-6 group at this timepoint (Figures 3A–E). However, no significant change of oxidative stress markers was found after 5 days of treatment.

## Discussion

The present study aimed to evaluate the neuroprotective effect of ω-3 PUFAs on LPS-induced inflammation and oxidative damage in the hippocampus of neonatal rats. We found that TLR4/NF-κB-mediated expression of proinflammatory cytokines and the level of oxidative damage in the hippocampus were significantly enhanced in response to systemic LPS administration. Treatment with ω-3 PUFAs improved all these parameters in the hippocampus, suggesting that ω-3 PUFAs may protect neonatal brain against infection-induced inflammation and oxidative stress. Viewing that DHA and EPA have shown neuroprotection against brain injury (32, 33), memory impairment (34, 35) and motor malfunction (36) in ischemic rats and mice, our next goal is to decipher the contribution of the major components of ω-3 PUFAs to neuroprotection in our LPS-challenge rat model.

In the present study, we used a single i.p. injection with LPS to trigger activation of inflammatory signaling and oxidative stress in the brain of neonatal rats. Our results showed that all the expression of TLR-4, NF-κB, and downstream proinflammatory factors, including TNF-α, IL-1β, and IL-6, was significantly upregulated in the hippocampal tissues in the LPS/saline group compared with the control group beginning on the day of LPS-challenge and continuing for 5 days. On day 1 or day 5 after LPS application, the TLR4, NF-κB, TNF-α, IL-1β, and IL-6 mRNA expression levels were significantly higher in the LPS group, the ω-6 group and the ω-3 group than in the control group (*P* < 0.05). The mRNA levels of TLR4, NF-κB, TNF-α, IL-1β and IL-6 in the ω-3 group were lower than those in the ω-6 group on days 1 and 5 (all *P* < 0.05). In contrast, the TLR4, NF-κB, TNF-α, IL-1β, and IL-6 mRNA expression levels in hippocampal tissues were higher in the ω-6 group than in the LPS group on days 1 and 5 (*P* < 0.05). These data suggest that ω-3 PUFAs, but not ω-6 PUFAs, may inhibit LPS-induced inflammatory response in neonatal rat brain.

Similarly, the activities of SOD and GSH were significantly decreased, whereas the amounts of MDA and GSSG were noticeably elevated in the LPS/saline group compared with the control group on the same day of LPS treatment. These results suggest that single systemic LPS administration is adequate to induce short-lasting inflammation response and oxidative stress in the neonatal rat brain, which is consistent with previous reports (20–22, 37–42). The activities of SOD and GSH in the hippocampal tissues were significantly higher in the ω-3 group than in the ω-6 and LPS groups on day 1 (both *P* < 0.05). Further, the amounts of MDA and GSSG in the hippocampus in the ω-3 group were significantly lower than those in the ω-6 and LPS groups on day 1 (both *P* < 0.05). In addition, the ratios of GSSG to GSH in the hippocampal tissues were significantly lower in the ω-3 group than in the ω-6 and LPS groups on day 1 (both *P* < 0.05). However, on day 5, the activity of SOD and GSH and the amounts of MDA and GSSG in the hippocampal tissues from the four groups were not significantly different (*P* > 0.05). These results suggest that ω-3 PUFAs may reduce oxidative stress induced by LPS in neonatal rat brain.

Both ω-3 and ω-6 PUFAs are essential FAs in the human diet (24). Accumulating evidence has demonstrated that ω-3 PUFAs are beneficial in a multitude of diseases, including autoimmune disorders, inflammatory diseases, and heart disease (43–47). The anti-inflammatory properties of ω-3 PUFAs are well-characterized in chronic inflammation-associated disorders, such as obesity, rheumatoid arthritis, coronary heart disease, and Crohn's disease (48–50). A diet rich in ω-6 PUFAs produce proinflammatory eicosanoids, whereas ω-3 PUFAs are able to decrease the production of proinflammatory cytokines and eicosanoids in various tissues and cells (51). Consistently, in this study, we observed that ω-6 PUFAs treatment further enhanced the expression of the TLR4/NF-κB signaling and downstream inflammation mediators induced by LPS. By contrast, ω-3 PUFAs exhibited anti-inflammatory effects based on their ability to inhibit the TLR4/NF-κB signaling, thereby decreasing the production of proinflammatory cytokines. Because proinflammatory cytokines, such as TNF-α and IL-1β, exert toxic effects on brain tissues through activating microglia and astrocytes and promoting accumulation of neutrophils, monocytes and lymphocytes (52, 53), our results suggest that ω-6 PUFAs may exacerbate, whereas ω-3 PUFAs may improve LPS-induced neuronal damage in newborn rats (31, 54, 55), However, we did not directly show the pathological alterations occurred in the rat brain (56) in this study, which should be addressed in the future.

In addition, macrophages and other immune cells generate excessive ROS upon exposure to LPS, resulting in decreased antioxidant capacity, overactive lipid peroxidation, and a disrupted redox balance (57). Premature brain is particularly vulnerable to redox imbalance (58, 59). To evaluate the effect of ω-3 PUFAs on LPS-induced oxidative damage in neonatal rat brain, we determined the amount of multiple oxidative stress markers in the hippocampus. The activities of SOD and GSH, which are important compounds for free radical removal, represent the state of the antioxidant system. On the other hand, products of oxidation processes, such as MDA and GSSG, are indirect indicators of the severity of oxidative damage (60). In this study, we observed that the SOD and GSH expression in the hippocampus of newborn rats were significantly decreased, whereas the amount of MDA and GSSG and the ratio of GSSG/GSH were remarkably increased in the LPS/ω-3 group compared with the LPS-saline group on the same day of LPS-challenge, suggesting that ω-3 PUFAs may reduce the damage caused by oxidative stress products in brain tissue. Opposite effects were observed in the LPS/ω-6 group, suggesting that ω-6 PUFAs may aggravate infection-induced neuronal injury. Although significant changes in inflammation-related genes lasted for at least 5 days after LPS-challenge, no significant difference was observed in the oxidative markers on the fifth day after LPS application. This finding suggest that inflammation and oxidative damage are not entirely correlated in the context of LPS, which requires further investigation.

Although the interaction between inflammation (their lipid mediator derivatives) and PUFAs is complex and poorly understood, EPA and DHA in ω-3 PUFAs are generally regarded to be anti-inflammatory and neuroprotective, to promote resolution of inflammation and to decrease pain in inflammatory conditions (36). By contrast, ω-6 PUFAs, including ARA, produce not only pro-inflammatory eicosanoids, but also lipid mediators that play an important role in inflammation resolution (61).

Hormesis, as elegantly explained in reviews (62), is featured with a biphasic dose response pattern (i.e., low dose stimulation and high dose inhibition). Although ω-3 PUFAs is extensively studied (44) and some showed a dose-dependent effect (63), it is so far not investigated in a specific hormetic approach as for *Ginkgo biloba* extract components (64) and some polyphenols (65, 66). Future study should be designed to explore its hermetic potential. Although ω-3 PUFAs modulated the transcription and translation of some vitagenes (67–69) in our animal model, such as SOD and GSSG/GSH, its antioxidant role in humans remains to be seriously tested.

Some of the drawbacks of the current study are that only one dose of each PUFAs and one age group of rats were used, one cannot possibly see any dosage effect required to observe hormesis (62) and link age with LPS susceptibility and PUFAs treatment, and only hippocampal tissues were collected, limiting opportunity to observe the systemic effect of LPS and PUFAs. Indeed, systemic LPS challenge could affect organs (kidney, lung, liver, and brain) to a different degree *via* different molecular mechanisms (70), and age is a critical factor to skew cytokine production pattern upon LPS treatment (71). Future work preferably should use a more elegant and sophisticated strategy to expand the current study.

## Conclusion

In conclusion, ω-3 PUFA supplementation may have neuroprotective effects against LPS-induced inflammation and oxidative damage in neonatal rats through downregulating the expression of TLR4/NF-κB-mediated proinflammatory cytokines and reducing oxidative stress, respectively. This finding may provide ω-3 PUFAs as potential therapeutic agents in protecting or alleviating infection-induced neonatal brain injury in preterm infants.

## Data Availability Statement

The datasets presented in this study can be found in online repositories. The names of the repository/repositories and accession number(s) can be found in the article/Supplementary Material.

## Ethics Statement

The animal study was reviewed and approved by Wenzhou Medical University, wydw2019-0501.

## Author Contributions

All authors listed have made a substantial, direct and intellectual contribution to the work and approved it for publication.

## Conflict of Interest

The authors declare that the research was conducted in the absence of any commercial or financial relationships that could be construed as a potential conflict of interest.
